# Supplementary material for: Effect of dietary treatment and fluid intake on the prevention of recurrent calcium stones and changes in urine composition: A meta-analysis and systematic review
Source: PLoS One. 2021 Apr 19;16(4):e0250257. doi: 10.1371/journal.pone.0250257 (PMC8055022; doi:10.1371/journal.pone.0250257)
Supplement: S1 Checklist — (DOC) [file pone.0250257.s001.doc]

| **Section/topic** | **#** | **Checklist item** | **Reported on page #** |
| --- | --- | --- | --- |
| **TITLE** | | |  |
| Title | 1 | Effect of dietary treatment and fluid intake on the prevention of recurrent calcium stones and changes in urine composition: a meta-analysis and systematic review | 1 |
| **ABSTRACT** | | |  |
| Structured summary | 2 | To perform a systematic review and meta-analysis of randomized controlled trials (RCTs) for investigating the effect of dietary treatment and fluid intake on the prevention of recurrent calcium stones and changes in urine composition.PubMed, Web of Science, Embase, EBSCO, and Cochrane Library databases (updated November 2020) were searched for studies with the following keywords: diet, fluid, recurrent, prevention, randomized controlled trials, and nephrolithiasis. The search strategy and study selection process was conducted by following the PRISMA statement. Six RCTs were identified for satisfying the inclusion criteria and enrolled in this meta-analysis. Our results indicate that the dietary intervention does not decrease the recurrence of calcium stone (RR = 1.15, 95% CI = 0.32–4.10; P = 0.83) with significant heterogeneity among the studies (I2 = 85%, P = 0.001). However, the fluid intake has a positive effect on prevention of recurrent stone formation (RR=0.39, 95% CI =0.19–0.80; P=0.01) with insignificant heterogeneity among the studies (I2 = 9%, P = 0.30). The different components of urine at baseline were reported in four studies. Upon reviewing the dietary therapy groups, it was found that there were no obvious changes in the 24-hour urine sodium, calcium, citrate, urea, and sulfate.Our study shows that the present dietary treatment containing low protein and high fiber does not reduce the recurrence rate of calcium stones. However, fluid intake has a significant effect on the reduction of recurrent calcium stones. | 2 |
| **INTRODUCTION** | | |  |
| Rationale | 3 | The previous meta-analysis includes only the recurrence rate and neglected the changes in urine compositions which makes the result unpersuasive and unclear [12]. Furthermore, new studies with more detailed data at high evidence level are reported. Thus, we performed this systematic review and meta-analysis of randomized controlled trials (RCTs) for investigating the effects of dietary treatment and fluid intake on the prevention of recurrent calcium stones and changes in urine composition. | 3 |
| Objectives | 4 | To perform a systematic review and meta-analysis of randomized controlled trials (RCTs) for investigating the effect of dietary treatment and fluid intake on the prevention of recurrent calcium stones and changes in urine composition. | 3 |
| **METHODS** | | |  |
| Protocol and registration | 5 | This study is not registered. | - |
| Eligibility criteria | 6 | (1) RCTs study design, (2) The patient had a history of urinary calcium stones and stone has been cleared by surgery, (3) the intervention was diet or water intake, (4) adequate reporting of data provided for analysis, and (5) availability of the full text. | 4 |
| Information sources | 7 | We systematically searched several databases including PubMed, Embase, Web of Science, EBSCO, and the Cochrane Library from the inception until November 2020 with the following keywords: diet, fluid, recurrent, prevention, randomized controlled trials, and nephrolithiasis. | 4 |
| Search | 8 | The article selection process was performed in accordance with the PRISMA guidelines. | 4 |
| Study selection | 9 | The reference lists of retrieved studies and relevant reviews were hand-searched, and the process mentioned above was repeatedly performed for ensuring that all eligible studies were included. | 4 |
| Data collection process | 10 | Data were independently extracted by two investigators. Discrepancies were resolved by consensus. | 4 |
| Data items | 11 | We assess Standard Mean differences (Std. MDs) with 95% confidence intervals (CIs) for continuous outcomes, and risk ratios (RR) with 95% CIs for dichotomous outcomes. Heterogeneity is evaluated using the I^2^ statistic, and I2 > 50% indicates significant heterogeneity. | 5 |
| Risk of bias in individual studies | 12 | We used the Jadad Scale to evaluate the quality of the study. | - |
| Summary measures | 13 | Risk ratios (RR) with 95% confidence intervals (CIs) were calculated for dichotomous outcomes. Heterogeneity was evaluated using the I2 statistic | 5 |
| Synthesis of results | 14 | I^2^ > 50% taken to indicate significant heterogeneity. | 5 |

Page 1 of 2

| **Section/topic** | **#** | **Checklist item** | **Reported on page #** |
| --- | --- | --- | --- |
| Risk of bias across studies | 15 | Two studies were considered to be of low quality due to the lack of blind method and detailed randomization methods while the other four studies were of high quality. | 5 |
| Additional analyses | 16 | Sensitivity analysis was performed for evaluating the influence of a single study on the overall estimate by omitting one study in turn or performing subgroup analysis. | 5 |
| **RESULTS** | | |  |
| Study selection | 17 | A total of 71 articles were initially identified from the database search. After the removal of duplicates, 45 articles were retained. Of these, 34 were excluded from analysis following the screening of the abstracts and titles, three were excluded as they were review articles, one was excluded because of insufficient data, and one was excluded because of the unavailability of the full text. Finally, six RCTs were identified as those satisfying the inclusion criteria and were finally enrolled in this meta-analysis | 5-6 |
| Study characteristics | 18 | Shown in the table 1. | 6 |
| Risk of bias within studies | 19 | - | - |
| Results of individual studies | 20 | Shown in the table 1. | 6 |
| Synthesis of results | 21 | dietary intervention does not decrease the recurrence of stone upon comparing with control groups (RR = 1.15, 95% CI = 0.32–4.10; P = 0.83) with significant heterogeneity among the studies (I2 = 85%, P = 0.001) (Fig 2). However, the fluid intake was found to have a positive effect on the prevention of stone recurrence (RR=0.39, 95% CI =0.19–0.80; P=0.01) with insignificant heterogeneity among the studies (I2 = 9%, P = 0.30, Fig 3). Three dietary groups reported the rate of withdrawal of patients. Though the result is statistically insignificant (RR = 0.76, 95% CI =0.59–0.98; P = 0.03) insignificant heterogeneity was observed among the studies (I2 = 0%, P = 0.81) | 6 |
| Risk of bias across studies | 22 | - | - |
| Additional analysis | 23 | Among all outcomes, the dietary intervention showed a significant heterogeneity (I2 = 85%, P = 0.001). A sensitivity analysis was performed to evaluate the stability of the results. After removing one study at a time, the heterogeneity showed I2 = 92%, 87%, and 81% indicating that the heterogeneity was stable. | 7 |
| **DISCUSSION** | | |  |
| Summary of evidence | 24 | The topical use EMLA is effective for reducing pain during EWSL, However, this analgesic effect is limited and does not reduce the use of analgesics | 10 |
| Limitations | 25 | Firstly, the characteristics of stone including stone size, position, composition, and severity of obstruction which may affect the pain of the procedure could not make subgroup to analysis. Secondly, more types of intraoperative analgesics is worth pondering in future study. Lastly, missing and unpublished data also led to bias in the true impact of EMLA. | 9-10 |
| Conclusions | 26 | In conclusion, the results of this systematic review showed that topical use EMLA is effective for reducing pain during EWSL, However, this analgesic effect is limited and does not reduce the use of analgesics | 10 |
| **FUNDING** | | |  |
| Funding | 27 | N/A | - |

*From:*  Moher D, Liberati A, Tetzlaff J, Altman DG, The PRISMA Group (2009). Preferred Reporting Items for Systematic Reviews and Meta-Analyses: The PRISMA Statement. PLoS Med 6(7): e1000097. doi:10.1371/journal.pmed1000097

For more information, visit: **www.prisma-statement.org**.

Page 2 of 2
