# Supplementary material for: Effect of dietary treatment and fluid intake on the prevention of recurrent calcium stones and changes in urine composition: A meta-analysis and systematic review
Source: PLoS One. 2021 Apr 19;16(4):e0250257. doi: 10.1371/journal.pone.0250257 (PMC8055022; doi:10.1371/journal.pone.0250257)
Supplement: S1 Table — (DOCX) [file pone.0250257.s002.docx]

**S1 Table for quality assessment in all the selected studies for systematic review and meta-analysis**

|  | Studies | Randomization  (0–2 points) | Blinding  (0–2 points) | Dropouts and withdrawals  (0–1 points) | Total score  (0–5 points) |
| --- | --- | --- | --- | --- | --- |
| 1 | Dussol 2008 | 2 | 2 | 1 | \| 5 \| \| --- \| |
| 2 | Sarica 2006 | 1 | 0 | 1 | 2 |
| 3 | Borghi 2002 | 2 | 0 | 1 | 3 |
| 4 | Kocvar 1999 | 1 | 0 | 1 | 2 |
| 5 | Hiatt 1996 | 2 | 1 | 1 | 4 |
| 6 | Borghi 1996 | 1 | 0 | 1 | 2 |
