# Supplementary material for: Effect of dietary treatment and fluid intake on the prevention of recurrent calcium stones and changes in urine composition: A meta-analysis and systematic review
Source: PLoS One. 2021 Apr 19;16(4):e0250257. doi: 10.1371/journal.pone.0250257 (PMC8055022; doi:10.1371/journal.pone.0250257)
Supplement: S1 Data — (DOCX) [file pone.0250257.s003.docx]

**Data for Effect of dietary treatment and fluid intake on the prevention of recurrent calcium stones and changes in urine composition: a meta-analysis and systematic review**

**Data Availability Statement:** All data used in this study are publicly accessible on PubMed, Embase, Web of Science, EBSCO, and the Cochrane library database via the DOIs included in the ‘References’ section of the paper.

**Data for recurrence rate of dietary interventions**

| Study | Dietary intervention | | Control | |
| --- | --- | --- | --- | --- |
|  | Recurrence | Total | Recurrence | Total |
| Dussol 2008 | 28 | 50 | 11 | 23 |
| Hiatt 1996 | 12 | 50 | 2 | 49 |

**Data of recurrence rate of liquid intake**

| Study | Fiuld intervention | | Control | |
| --- | --- | --- | --- | --- |
|  | Recurrence | Total | Recurrence | Total |
| Borghi 1996 | 12 | 99 | 27 | 100 |
| Sarica 2006 | 1 | 12 | 5 | 9 |

**Data of withdraw rate**

| Study | Dietary intervention | | Control | |
| --- | --- | --- | --- | --- |
|  | Recurrence | Total | Recurrence | Total |
| Borghi 2002 | 6 | 60 | 7 | 60 |
| Dussol 2008 | 55 | 115 | 37 | 60 |
| Hiatt 1996 | 8 | 50 | 13 | 49 |

**Data of urinary compositions variables in baseline and the long term follow up**

| Study | Follow up | Method | Urine volume  (L/day) | | Sodium  (mmol/day) | | Calcium  (mmol/day) | | Oxalate  (mmol/day) | | Citrate  (mmol/day) | | Urea  (mmol/day) | | Sulfate  (mmol/day) | | Relative oxalate saturation | |
| --- | --- | --- | --- | --- | --- | --- | --- | --- | --- | --- | --- | --- | --- | --- | --- | --- | --- | --- |
|  |  |  | Baseline | Result | Baseline | Result | Baseline | Result | Baseline | Result | Baseline | Result | Baseline | Result | Baseline | Result | Baseline | Result |
| Dussol | 3 year | Low protein | 1.8±0.6 | 1.9±0.8 | 149±44 | 171±71 | 6.8±3.1 | 7.0±3.5 | 0.30±0.1 | 0.29±0.1 | 2.9±1.9 | 2.9±1.5 | 381±95 | 359±135 | 4.3±1.9 | 3.1±2.4 |  |  |
|  |  | High fiber | 2.0±0.7 | 1.8±0.6 | 163±58 | 154±55 | 6.9±3.7 | 7.0±3.5 | 0.31±0.2 | 0.32±0.1 | 3.3±3.2 | 2.1±1.2 | 354±93 | 361±117 | 4.6±2.8 | 4.8±3.6 |  |  |
| Borghi | 3 year | Low protein  Low salt | 1.9±6.7 | 2.1±5.2 | 205±64 | 127±66^b^ | 7.0±3.4 | 6.6±2.4 | 0.42±0.1 | 0.33±0.1^b^ |  |  | 505±142 | 447±113 | 2.8±0.8 | 2.5±0.6 | 6.7±4.5 | 4.5±2.9^a^ |
| Kocvar | 3 year | Special diet | 2.4±6.5 | 2.4±6.5 |  |  | 5.1±2.4 | 6.4±2.8^b^ | 0.35±0.2 | 0.42±0.2^a^ | 3.0±1.5 | 3.2±1.9 |  |  |  |  |  |  |
| Borghi | 5 year | Fluid intake | 1.1±0.2 | 2.6±0.7^b^ |  |  |  |  |  |  |  |  |  |  |  |  | 1.8±1.7 | 1.2±1.0^b^ |
