# Supplementary material for: Effect of dietary treatment and fluid intake on the prevention of recurrent calcium stones and changes in urine composition: A meta-analysis and systematic review
Source: PLoS One. 2021 Apr 19;16(4):e0250257. doi: 10.1371/journal.pone.0250257 (PMC8055022; doi:10.1371/journal.pone.0250257)
Supplement: S1 Search strategy — (DOCX) [file pone.0250257.s004.docx]

S1 search strategy for Electronic database

| **No** | **Database** | **Date and time accessed** | **Search query** | **hits** |
| --- | --- | --- | --- | --- |
|  | PUBMED | 31/08/2019, 08:49 am | ("diet"[MeSH Terms] OR "diet"[All Fields] OR ("fluid"[All Fields] OR "fluid s"[All Fields] OR "fluids"[All Fields])) AND ("recurrance"[All Fields] OR "recurrence"[MeSH Terms] OR "recurrence"[All Fields] OR "recurrences"[All Fields] OR "recurrencies"[All Fields] OR "recurrency"[All Fields] OR "recurrent"[All Fields] OR "recurrently"[All Fields] OR "recurrents"[All Fields]) AND ("prevent"[All Fields] OR "preventability"[All Fields] OR "preventable"[All Fields] OR "preventative"[All Fields] OR "preventatively"[All Fields] OR "preventatives"[All Fields] OR "prevented"[All Fields] OR "preventing"[All Fields] OR "prevention and control"[MeSH Subheading] OR ("prevention"[All Fields] AND "control"[All Fields]) OR "prevention and control"[All Fields] OR "prevention"[All Fields] OR "prevention s"[All Fields] OR "preventions"[All Fields] OR "preventive"[All Fields] OR "preventively"[All Fields] OR "preventives"[All Fields] OR "prevents"[All Fields]) AND ("randomized controlled trial"[Publication Type] OR "randomized controlled trials as topic"[MeSH Terms] OR "randomized controlled trials"[All Fields] OR "randomised controlled trials"[All Fields]) AND ("nephrolithiasis"[MeSH Terms] OR "nephrolithiasis"[All Fields]) | 26 |
|  | Embase | 30/08/2019, 04:51 pm | ("diet"[MeSH Terms] OR "diet"[All Fields] OR ("fluid"[All Fields] OR "fluid s"[All Fields] OR "fluids"[All Fields])) AND ("recurrance"[All Fields] OR "recurrence"[MeSH Terms] OR "recurrence"[All Fields] OR "recurrences"[All Fields] OR "recurrencies"[All Fields] OR "recurrency"[All Fields] OR "recurrent"[All Fields] OR "recurrently"[All Fields] OR "recurrents"[All Fields]) AND ("prevent"[All Fields] OR "preventability"[All Fields] OR "preventable"[All Fields] OR "preventative"[All Fields] OR "preventatively"[All Fields] OR "preventatives"[All Fields] OR "prevented"[All Fields] OR "preventing"[All Fields] OR "prevention and control"[MeSH Subheading] OR ("prevention"[All Fields] AND "control"[All Fields]) OR "prevention and control"[All Fields] OR "prevention"[All Fields] OR "prevention s"[All Fields] OR "preventions"[All Fields] OR "preventive"[All Fields] OR "preventively"[All Fields] OR "preventives"[All Fields] OR "prevents"[All Fields]) AND ("randomized controlled trial"[Publication Type] OR "randomized controlled trials as topic"[MeSH Terms] OR "randomized controlled trials"[All Fields] OR "randomised controlled trials"[All Fields]) AND ("nephrolithiasis"[MeSH Terms] OR "nephrolithiasis"[All Fields]) | 7 |
|  | Web of science | 30/08/2019, 07:15 pm | ("diet"[MeSH Terms] OR "diet"[All Fields] OR ("fluid"[All Fields] OR "fluid s"[All Fields] OR "fluids"[All Fields])) AND ("recurrance"[All Fields] OR "recurrence"[MeSH Terms] OR "recurrence"[All Fields] OR "recurrences"[All Fields] OR "recurrencies"[All Fields] OR "recurrency"[All Fields] OR "recurrent"[All Fields] OR "recurrently"[All Fields] OR "recurrents"[All Fields]) AND ("prevent"[All Fields] OR "preventability"[All Fields] OR "preventable"[All Fields] OR "preventative"[All Fields] OR "preventatively"[All Fields] OR "preventatives"[All Fields] OR "prevented"[All Fields] OR "preventing"[All Fields] OR "prevention and control"[MeSH Subheading] OR ("prevention"[All Fields] AND "control"[All Fields]) OR "prevention and control"[All Fields] OR "prevention"[All Fields] OR "prevention s"[All Fields] OR "preventions"[All Fields] OR "preventive"[All Fields] OR "preventively"[All Fields] OR "preventives"[All Fields] OR "prevents"[All Fields]) AND ("randomized controlled trial"[Publication Type] OR "randomized controlled trials as topic"[MeSH Terms] OR "randomized controlled trials"[All Fields] OR "randomised controlled trials"[All Fields]) AND ("nephrolithiasis"[MeSH Terms] OR "nephrolithiasis"[All Fields]) | 325 |
|  | EBSCO |  | ("diet"[MeSH Terms] OR "diet"[All Fields] OR ("fluid"[All Fields] OR "fluid s"[All Fields] OR "fluids"[All Fields])) AND ("recurrance"[All Fields] OR "recurrence"[MeSH Terms] OR "recurrence"[All Fields] OR "recurrences"[All Fields] OR "recurrencies"[All Fields] OR "recurrency"[All Fields] OR "recurrent"[All Fields] OR "recurrently"[All Fields] OR "recurrents"[All Fields]) AND ("prevent"[All Fields] OR "preventability"[All Fields] OR "preventable"[All Fields] OR "preventative"[All Fields] OR "preventatively"[All Fields] OR "preventatives"[All Fields] OR "prevented"[All Fields] OR "preventing"[All Fields] OR "prevention and control"[MeSH Subheading] OR ("prevention"[All Fields] AND "control"[All Fields]) OR "prevention and control"[All Fields] OR "prevention"[All Fields] OR "prevention s"[All Fields] OR "preventions"[All Fields] OR "preventive"[All Fields] OR "preventively"[All Fields] OR "preventives"[All Fields] OR "prevents"[All Fields]) AND ("randomized controlled trial"[Publication Type] OR "randomized controlled trials as topic"[MeSH Terms] OR "randomized controlled trials"[All Fields] OR "randomised controlled trials"[All Fields]) AND ("nephrolithiasis"[MeSH Terms] OR "nephrolithiasis"[All Fields]) | 143 |
|  | Cochrane libirary |  | ("diet"[MeSH Terms] OR "diet"[All Fields] OR ("fluid"[All Fields] OR "fluid s"[All Fields] OR "fluids"[All Fields])) AND ("recurrance"[All Fields] OR "recurrence"[MeSH Terms] OR "recurrence"[All Fields] OR "recurrences"[All Fields] OR "recurrencies"[All Fields] OR "recurrency"[All Fields] OR "recurrent"[All Fields] OR "recurrently"[All Fields] OR "recurrents"[All Fields]) AND ("prevent"[All Fields] OR "preventability"[All Fields] OR "preventable"[All Fields] OR "preventative"[All Fields] OR "preventatively"[All Fields] OR "preventatives"[All Fields] OR "prevented"[All Fields] OR "preventing"[All Fields] OR "prevention and control"[MeSH Subheading] OR ("prevention"[All Fields] AND "control"[All Fields]) OR "prevention and control"[All Fields] OR "prevention"[All Fields] OR "prevention s"[All Fields] OR "preventions"[All Fields] OR "preventive"[All Fields] OR "preventively"[All Fields] OR "preventives"[All Fields] OR "prevents"[All Fields]) AND ("randomized controlled trial"[Publication Type] OR "randomized controlled trials as topic"[MeSH Terms] OR "randomized controlled trials"[All Fields] OR "randomised controlled trials"[All Fields]) AND ("nephrolithiasis"[MeSH Terms] OR "nephrolithiasis"[All Fields]) | 3 |
|  | Total | 504 | | |
|  | After removal of duplicates | 245 | | |
|  | After assessment of title and abstract | 11 | | |
|  | Finally included | 6 | | |
