# Supplementary material for: Effect of dietary treatment and fluid intake on the prevention of recurrent calcium stones and changes in urine composition: A meta-analysis and systematic review
Source: PLoS One. 2021 Apr 19;16(4):e0250257. doi: 10.1371/journal.pone.0250257 (PMC8055022; doi:10.1371/journal.pone.0250257)
Supplement: Attachment — Submitted filename: Response to Reviewers.docx [file pone.0250257.s005.docx]

**Dear Editor**

**Thank you very much for giving us an opportunity to revise our manuscript. We appreciate the editor and reviewers very much for their constructive comments and suggestions on our manuscript entitled “Effect of dietary treatment and fluid intake on the prevention of recurrent calcium stones and changes in urine composition: a meta-analysis and systematic review”. We have studied reviewers’comments carefully. According to the reviewers’ detailed suggestions, we have made a careful revision on the original manuscript. All revised portions are marked in the revised manuscript which we would like to submit for your kind consideration. Due to the loss of original search data, we re-searched the electronic database. Though the process changed，the result do not changed. We have rewritten this part and upload the search strategy. We appreciate for Editors/Reviewers’ warm work earnestly and hope that the correction will meet with approval. Once again, thank you very much for your comments and suggestions. We hope that the revisions in the manuscript and our accompanying responses will be sufficient to make our manuscript suitable for publication in your journal.**

**Kind regards.**

**Corresponding author : Wei wuran**

**E-mail: wzh2019kb@163.com**

Comments for the investigators:

Reviewer #1：
1. The Introduction includes the statement that “medication has a therapeutic effect on the prevention of recurrent stone formation by reducing the tubular reabsorption or increasing the intestinal reabsorption of calcium.” This is incorrect, and in fact these changes in renal or intestinal calcium handling would actually lead to higher urine calcium excretion. The effect of thiazide is to increase renal calcium reabsorption, in addition to a modest effect to decrease gut calcium absorption. The effect of citrate is multifactorial and includes its ability to chelate calcium in the urine, and to poison calcium crystal surfaces; it may also lower urine calcium by increasing renal calcium reabsorption.

**Response:** We are very sorry for the mistake and appreciated for the reviewer’s correction. We have corrected this part as the comment (Currently, medication has a therapeutic effect on the prevention of recurrent stone formation by increasing renal calcium reabsorption, decreasing gut calcium absorption, chelating calcium in the urine or poisoning calcium crystal surfaces.).

2. The authors used the Jadad scale to assess for study quality. The 3 items in the scale assess randomization (2 possible points), blinding (2 possible points), and accounting for all patients (1 point). The authors need to justify use of this scale for long-term dietary/fluid studies in which blinding is not attempted or likely to succeed. If possible, a more appropriate scale should be used which was designed for this purpose.

**Response:** Thanks for this professional comments. We have changed the assessment tool. We used the risk bias tool of Cochrane Handbook for Systematic Reviews of Intervention which is more evidence based and we changed this part correspondingly.

3. The diet studies are not strictly comparable with respect to the interventions carried out. Diet alone is not an intervention, rather the specific type of diet interventions is what is being tested. For background: The 4 diet studies comprise 9 study arms. Three studies have 2 arms, while Dussol has three (control and 2 interventions). The control arms in three studies (Hiatt, Kocvara, and Dussol) are somewhat similar (normal calcium intake, encourage fluids) while the interventions vary. Two of these studies specifically study animal protein and fiber in the diet. In Hiatt, the intervention is low animal protein, increased fruits and vegetables, and bran; in Dussol the 2 intervention arms compare low animal protein (arm 1) with increased fruits and vegetables and whole grain (arm 2). Patient numbers are quite small in Dussol due to drop outs. In Hiatt, the control arm does better with respect to stone recurrence, while in Dussol the three arms all have a very high recurrence rate; both studies follow patients for about 4 years. Overall, it seems fair to say that low animal protein diet, with or without high fiber (or high fiber diet alone), does not prove to lower stone recurrence better than a diet with high fluid and normal calcium intake alone, based on these 2 studies. The recurrence rates per year do vary a lot in these studies, and the authors do not attempt to report them. I suggest that the authors use only these 2 studies to evaluate the effect of protein restriction and fiber. The diet studies by Kocvara and by Borghi are not so readily compared with Hiatt and Dussol. While the control arm of Kocvara is similar, what is being tested in the intervention arm is not a specific diet, but rather the concept that diet prescription based on metabolic workup, rather than generic advice, is beneficial, and the study results bear this out. Thus this study does not really test a specific diet per se, but rather whether diets should be tailored or generic. This study does not really belong in this analysis, as the type of dietary advice is unknown in the intervention group, being individualized to each patient.

**Response：**Thanks for this comment. We have removed the Kocvara et al. for the analysis for recurrent rate and only take Hiatt and Dussol into primary outcome as comment. And the result did not changed as the new result showed that dietary intervention does not decrease the recurrence of stone upon comparing with control groups (RR = 2.32, 95% CI = 0.42–12.85; P = 0.34) with significant heterogeneity among the studies (I2 = 81%, P = 0.02). Meanwhile, we keep Kocvara et al in our study because the data of changes in the urine compositions. We have corrected this part in our study accordingly.

4. The diet study by Borghi compares low calcium diet (a unique diet arm which differs markedly from the control arms in other studies) to a diet with low sodium intake (not studied in any of the other studies), along with normal calcium intake, and modestly low animal protein intake. The fact that sodium restriction is the main intervention is clearly shown in Table 2, in which urine Na clearly falls on follow-up, while urine urea does not. The authors should not analyze this study as a low protein trial, because it was sodium restriction which was the major intervention. At 5 years (the study endpoint) the recurrence rates are much lower in the low sodium arm compared to low calcium diet This study is not a simple low protein trial, and should not be included with the 2 explicitly low protein diet trials. In addition, the patient group in this study differs from the other trials, in that idiopathic hypercalciuria, rather than only calcium stone formation, was an explicit study inclusion criteria. The authors should rectify the errors in analysis of this trial, and analyze it as a unique study

**Response:** This is a very good comment. Sorry for ignorance of the effect of a low-salt diet on changes in the composition of urine in Borghi et al. study. And we have added the low salt diet in table two. And we have corrected the result part and described uniqueness of this study.

5. The paragraph titled Primary Outcome in results describes an analysis which seems to include all the diet studies (ref 17, 20-22) but Figure 2 shows only Dussol, Hiatt, and Kocvar.

**Response:** Sorry for the mistake we made. Though there are four studies reported the diet method, only three study reported the recurrence rate. Actually, Borghi et al. just reported the changes of urinary composition thus only three studies were included into the analysis (ref 16,19,20; Figure 2) and we have corrected this part.

6.Overall, the analysis does not compare like to like, lumping non-comparable studies together, Its handling of the Borghi diet trial is particularly unclear, and therefore the conclusion that dietary intervention does not decrease recurrence of calcium stone does not appear to be valid. A more limited conclusion, that low protein diet, with or without increased fiber, has not proven to lower calcium stone recurrence, may be justified.

**Response:** Thanks for this important comment. After re-examining our conclusions, we realized that they were too general to be drawn. Therefore, the limited conclusion is needed and we have correct according to this comments.

Reviewer #2:

1.Firstly, authors should emphasize that their study investigates as a primary outcome stone RECURRENCE per se as opposed to stone RISK FACTORS. This is important.

**Response:** It is a very good comment which may make our study clearer. After careful re-read for our study, we changed the description like “increase the risk of stone” into “increase the recurrence of stone”; “reduced risk or no relation for stone formation” into “reduced recurrence or no relation for stone formation” to emphasize the primary outcome stone recurrence according to comment. Meanwhile, our introduction and conclusion part also emphasize the aim of this study is to “investigating the effects of dietary treatment and fluid intake on the prevention of recurrent calcium stones”.

2.Second, the findings which refute popular views are equally important, namely that diet does not affect recurrence, that diet does not affect urine composition and that the latter does not affect the former. These are new and provocative findings and will initiate a re-think among stone researchers. Indeed, authors can challenge readers by posing a question about the clinical value of dietary interventions in the first place. Yes, this will be controversial, but that's what differentiates this study from others.

**Response:** Thank you for your appreciation and this very good comment on revision. We reorganized part of introduction and add this sentence “Previous studies have shown that formation of a renal stone is closely related to dietary regimes [12, 13]. Nevertheless, an evidence-based study of clinical value in dietary therapy in urinary stone recurrence is still lacked. Furthermore, new studies with more detailed data at high evidence level are reported.” With this sentence, we pose a question about the clinical value of dietary interventions in the first place and differentiates this study from others as the comments.

**Acknowledgment:** Our deepest gratitude goes to the reviewers and editors for their careful work and thoughtful suggestions that have helped improve this paper substantially. We also thanks for both their meaningful comments and recognition to our study again.
