# Supplementary material for: Effect of dietary treatment and fluid intake on the prevention of recurrent calcium stones and changes in urine composition: A meta-analysis and systematic review
Source: PLoS One. 2021 Apr 19;16(4):e0250257. doi: 10.1371/journal.pone.0250257 (PMC8055022; doi:10.1371/journal.pone.0250257)
Supplement: Attachment — Submitted filename: Response to reviewer.docx [file pone.0250257.s006.docx]

Reviewer #1: 
1. In the Introduction, the authors state that “..an evidence-based study of clinical value in dietary therapy in urinary stone recurrence is still lacked”. It is unclear why the authors make this statement, as they are reviewing a number of evidence-based studies. What do they feel is lacking?

**Response：**This is a very good comment. What we mean is higher evidence-based study like systematic review and meta-analysis and we have changed this part. We have changed the vague expression and thank you for your correction.

2. In the section on selection criteria, they specify that the in the studies they selected “patient had stone that has been cleared by surgery”. This is incorrect. The studies they are using do not require surgical removal of stones, only stone passage (or removal) or in some cases visualization on Xray.

**Response：**Thanks for comment. We have checked our origin study design and found the mistake in our description. The inclusion criteria is patient had a stone history and was diagnosed by surgical removal, stone passage, or by imaging systems. And we correct this part in our study.

3. In the section on Primary outcome, they state that 5 of the studies reported stone recurrence, omitting the Borghi trial of low sodium diet. This is incorrect. That study does report stone recurrence: “Twenty-three of the 60 men on the low-calcium diet and 12 of the 60 on the normal-calcium, low protein, low-salt diet had recurrences. The cumulative incidence of recurrent stones in the two groups is shown in Figure 2. The relative risk of a recurrence among the men in the normal-calcium, low-protein, low-salt group, as compared with the men in the low calcium group, was 0.49 (95 percent confidence interval, 0.24 to 0.98; P=0.04).” This data should be added to the section on primary outcome.

**Response：**Thanks for this very good comment. We ignore the result of Borghi trial and made an incomplete conclusion. And we have added the Borghi trail result into the primary outcome. And correcting the our related conclusion for diet intervention into two part：1. Low protein with or without high fiber do not affect the recurrence； 2. normal calcium, low sodium diet did reduce recurrence rate, as comment. Therefore, our conclusions are completer and more accurate.

4. The authors continue to refer to “diet method” as the intervention being studied. This is incorrect. The authors need to specify what type of diet is being evaluated. In the case of Hiatt and Dussol, it is low protein with or without high fiber. They should modify the statement in “Primary outcome: recurrence rate” to make clear that this type of diet did not reduce stone recurrence rate. They should also make clear that the Borghi study of normal calcium, low sodium diet did reduce recurrence rate. They should also modify Figure 3 accordingly, although given that there is only one study of low Na, normal Ca diet it is not clear that Borghi can be added to the plot. However, the legend should make clear that only trials of low protein with or without high fiber are included.

**Response：**Thanks for this comment. Hiatt and Dussol study show low protein with or without high fiber do not affect the recurrence. And Borghi study shows normal calcium, low sodium diet did reduce recurrence rate. We changed the primary result, figure legend and conclusion accordingly.

**5**. The authors state in the Discussion that changes in diet do not affect recurrence. This is incorrect. Low protein diet does not affect recurrence, while low Na, normal Ca diet has a marked effect on recurrence. This statement must be changed.

**Response：**Thanks for this detailed comment. This statement is too general which is incorrect, and we have corrected the statement correspondingly as comment in discussion part (Second paragraph; line 2) and conclusion part.

**Acknowledgment:** Thanks again. Our deepest gratitude goes to the you for your careful work and detailed comment that have helped improve the quality of our study.

Reviewer #2: The main interest in this study is that its findings are contrary to popular dogma. As such it is likely to attract a lot of attention.

**Acknowledgment:** Thank you for your recognition of our article. At the same time, we also deeply thank you for your careful and detailed review work. It is your very detailed and professional comments that make our articles more scientific and more complete.
